# ASSESSMENT OF THE LATE EFFECTS ON BONES AND ON BODY COMPOSITION OF CHILDREN AND ADOLESCENTS TREATED FOR ACUTE LYMPHOCYTIC LEUKEMIA ACCORDING TO BRAZILIAN PROTOCOLS

**DOI:** 10.1590/1984-0462/;2017;35;1;00005

**Published:** 2017-02-20

**Authors:** Poliana Cristina Carmona Molinari, Henrique Manoel Lederman, Maria Lucia de Martino Lee, Eliana Maria Monteiro Caran

**Affiliations:** a Instituto de Oncologia Pediátrica, Grupo de Apoio ao Adolescente e à Criança com Câncer (IOP/GRAACC), Universidade Federal de São Paulo (UNIFESP), São Paulo, SP, Brasil.; b Departamento de Radiologia, IOP/GRAACC/UNIFESP, São Paulo, SP, Brasil.

**Keywords:** Leukemia, lymphoblastic, Bone density/drug therapy, Bone mineral density/radiation effects

## Abstract

**Objective:**

To evaluate the impact of therapy on bone mineral density (BMD) and body composition in survivors of acute lymphoblastic leukemia (ALL) treated in accordance with Brazilian protocols by the Brazilian Cooperative Group of Treatment of Lymphoblastic Leukemia in Childhood (GBTLI) LLA-93 and LLA-99.

**Methods:**

A cross-sectional study with 101 patients was performed. BMD and body composition were evaluated using bone densitometry and were interpreted according to the age group and the reference population. Values between -1.1 and -1.9 in the group of children under 20 years were considered as risk group for low BMD z-scores. BMD values were compared to clinical characteristics, treatment received and body composition. A chi-square test, Fisher’s exact test, likelihood ratio and Student’s t-test were applied, with a 5% significance level.

**Results:**

The patients presented a frequency of fractures of 2%, of osteonecrosis, 2%, and of low BMD, 2.9%. In the group of 79 patients under 20 years of age, three had low BMD. The 16 that presented risk for low BMD, demonstrated lower valutes in lumbar vertebrae L1-L4 (*p*=0.01) and whole body (*p*=0.005), and smaller values of lean body mass (*p*=0.03). In the group of 22 patients over 20 years of age, ten had osteopenia.

**Conclusions:**

The low impact of treatment on BMD of this study confirms the concept that the bone mass gain occurs with increasing age and that the treatment does not influence the process. The population at risk for low BMD values presented lower bone mass values and could benefit from a long-term monitoring for possible bone toxicity.

## RESUMO

**Objetivo:**

Avaliar o impacto da terapia sobre a densidade mineral óssea (DMO) e composição corporal em sobreviventes da leucemia linfoide aguda (LLA), tratados de acordo com os protocolos brasileiros do Grupo Cooperativo Brasileiro de Tratamento de Leucemia Linfoide Aguda na Infância (GBTLI), LLA-93 e LLA-99.

**Métodos:**

Em estudo transversal com 101 pacientes, avaliaram-se a composição corporal e a DMO por meio da densitometria óssea, interpretando-a conforme a faixa etária e a população de referência. Foi considerado grupo de risco para baixa DMO valores de z-escore entre -1,1 e -1,9 no grupo dos menores de 20 anos. Compararam-se os valores da DMO com características clínicas, tratamento recebido e composição corporal. Foram utilizados os testes qui-quadrado, exato de Fisher, razão de verossimilhança e *t* de Student, com nível de significância de 5%.

**Resultados:**

Foram encontradas 2% de fraturas, 2% de osteonecrose e 2,9% de baixa DMO. No grupo de pacientes com menos de 20 anos, três apresentaram baixa DMO. Os 16 pacientes com risco para baixa DMO exibiram menores valores em vértebras lombares L1-L4 (*p*=0,01), corpo total (*p*=0,005) e valores mais baixos de massa magra (*p*=0,03). No grupo de 22 pacientes com mais de 20 anos, dez demonstraram osteopenia.

**Conclusões:**

O baixo impacto do tratamento sobre a DMO neste estudo ratifica o conceito de que o ganho de massa óssea ocorre com o aumento da idade e que o tratamento não influencia tal processo. A população de risco para baixa DMO demonstrou valores menores de massa óssea, podendo beneficiar-se de um acompanhamento em longo prazo para uma possível toxicidade óssea.

## INTRODUCTION 

The therapeutic progress obtained in the treatment of children with acute lymphocytic leukemia (ALL) over the past 50 years has resulted in cure rates of around 80%, culminating in an impactful increase in the number of survivors. This population represents a group with increased risk for comorbidity related to treatment and the disease itself.^1^ ^.^ ^2^ Changes in bone metabolism and body composition are considered important adverse late effects and represent a significant cause of morbidity in this population, through pain, fractures, decrease of bone mineral density (BMD) and chronic impairment of bone function.^3^ ^.^ ^4^ Exposure to corticosteroids, methotrexate, mercaptopurine and radiation, associated with low calcium intake, decreased physical activity, and obesity are some of the factors that lead to low BMD.^5^ ^,^ ^6^ ^,^ ^7^ Although patients treated for ALL can recover lost bone mass during the post-treatment period, a percentage of them will not reach their maximum BMD acquisition potential, presenting significant bone deficit.^1^

Bone densitometry is the method of choice for evaluating bone mineral density and body composition. However, it must be performed and interpreted in accordance with the pediatric references for each population, whose norms have been previously published.^8^ ^,^ ^9^ ^,^ ^10^

As such, the goal of this study was to evaluate the impact of chemotherapy and radiotherapy as adverse late effects of BMD in children and adolescents treated for ALL in accordance with Brazilian protocols, through the use of dual-energy X-ray absorptiometry (DXA).

## METHOD

This is a cross-sectional retrospective study with a convenience sample that included 242 patients with ALL that had been treated in accordance with the Brazilian Cooperative Group of Treatment of Lymphoblastic Leukemia in Childhood (Grupo Cooperativo Brasileiro para Tratamento da LLA na Infância - GBTLI), protocols LLA-93 and LLA-99. These patients were treated at the Institute of Pediatric Oncology (IOP) of the *Child and Adolescent Cancer Support Group* (GRAACC) at the Universidade Federal de São Paulo (UNIFESP), São Paulo (SP), Brazil.

The study included patients from both sexes who received such treatment from 1994 to 2006 and were over 5 years old at the time of data collection. At the time of the study, they were in regular clinical follow-up at the outpatient clinic and at pediatric endocrinology. All of the patients were in the first complete clinical remission, which is defined as the lack of disease in peripheral blood, in bone marrow, and in extramedullary compartments such as spinal fluid or testicles. Patients in the following situations were excluded from the study: those with concurrent chronic conditions that could affect bone growth (including kidney or liver disease, as well as other immunological and endocrinological diseases); those who had not been under regular monitoring at the outclinics mentioned above for at least three years; those with physical disabilities; smokers and alcoholics; recipients of bone marrow or solid organ transplants; pregnant patients; those presenting recurrence of ALL during or after treatment; those with Down syndrome and those who had made use of exogenous growth hormones in the past two years. Finally, patients under the following conditions were removed from the study: those who did not return for the examinations or who decided to abandon the study and those who could not be reached by phone or by letter due to outdated contact information. This population was not statistically significant if compared to the population investigated.

This study was approved by the Research Ethics Committee of the Medical School at the Universidade Federal de São Paulo (UNIFESP) under number 1.618/10. Patients and their parents (or legal guardians) were informed about the study and its goals, and agreed to sign an informed consent form and an agreement form.

Data collection took place between February 2011 and January 2013 and included the following information: demographic data, previous history of the disease and of cancer treatment (including the patients’ risk rating and whether they had undergone cranial radiotherapy, accumulated doses of prednisone, dexamethasone and chemotherapeutic agents), laboratory profile (creatinine serum, calcium, phosphorus, magnesium, alkaline phosphatase, thyroid and parathyroid hormones and vitamin D) and bone densitometry.

Demographic data included date of birth, current age and age at diagnosis, sex, race and time elapsed after treatment. Clinical characteristics related to the disease and treatment were obtained through a review of medical records, namely: symptoms of bone involvement at diagnosis; initial leukocyte count; immunophenotypical classification of ALL; occurrence of central nervous system (CNS) involvement at diagnosis; chemotherapy protocol received; risk classification; cumulative doses of prednisone, dexamethasone, methotrexate, alkylating agents and mercaptopurine; need for prophylactic radiotherapy; presence of bone toxicity during treatment; and time outside of therapy.

Anthropometric and pubertal development data was obtained during the physical examination at the outpatient endocrinology clinic. A mechanical balance (Filizola^®^) was used to measure weight, and height was measured using a wall-mounted stadiometer (Tonelli^®^), allowing for the computation of the patients’ body mass index (BMI). Nutritional diagnosis was made based on the parameters of the World Health Organization (WHO), through BMI values.

BMD measurement was performed at the anteroposterior projection on the lumbar spine segment L1-L4, whole body and femur, through DXA, using the Lunar DPX (GE Lunar Corporation^®^) instrument. All of the tests were analyzed by a single radiologist through the Encore software and interpreted according to age, sex and the software’s reference database. For patients under 20 years old, z-score levels under -2 standard deviations (SD) were considered as having low BMD values. Hypothetically, values between -1.9 and -1.1, conceptualized as normal by the literature, were considered as being at risk for low BMD. For patients above 20 years old, the T-score was calculated and compared with patterns of young adults. In this population, a T-score lower than -2.5 SD was considered as a case of osteoporosis. In the same fashion, a T-score between -1 and -2.5 SD indicated osteopenia. BMD values were matched against clinical characteristics and treatment, in addition to body composition.

Data was stored in spreadsheets, and all statistical analyses were performed through the use of the Statistical Package for Social Sciences (SPSS version 15.0) for Microsoft Windows. The following were treated as independent variables: gender, race, age at diagnosis, current age, time outside therapy, initial leukocyte count, CNS infiltration at time of diagnosis, immunophenotyping, risk classification, treatment received, cumulative doses of chemotherapy and corticosteroids, pubertal stage of Tanner, anthropometric data, body composition, and BMD values and their SD values. BMD classification values were treated as dependent variables: Normal BMD, low BMD and risk of low BMD for patients under 20 years; and normal BMD, osteopenia and osteoporosis for those over 20 years of age. Quantitative variables were expressed through arithmetic means and SD values. The matching of BMD values with categorical variables was performed using the chi-square test, Fisher’s exact test and the likelihood ratio. The matching of BMD values with numerical variables was performed using *t-*tests. The significance level was set at 5%.

## RESULTS

Out of the 242 patients treated for ALL, 76 died due to various causes and 65 of them were excluded from the study due to one or more predetermined criteria. This led to a total of 101 patients, 59.4% of which were female and 77.2% were white. The mean age of the patients was 17.2±4.9 years. With regard to characteristics at the time of diagnosis, the mean age was 5.2 ± 3.6 years, and 94% of patients had an immunophenotypical classification of precursor B-cell ALL. 78.2% of the patients presented an initial leukocyte count lower than 50,000 cells/mm^3^, and CNS infiltration was diagnosed in nine patients.

The GBTLI LLA-93 protocol was used in 44 patients and the GBTLI LLA-99 in 57 patients, 54.5% of which were classified as low risk. Treatment-related characteristics are shown in Table 1. All laboratory profiles showed values in agreement with the reference parameters for calcium, phosphorus, magnesium, serum creatinine and thyroid hormones; 16 patients presented alkaline phosphatase values above the reference for their age and gender; and 23 (23.4%) presented vitamin D values lower than 20 ng/dL.

**Table 1:** Characteristics of the treatment of lymphoblastic leukemia according to the protocols of the Brazilian Cooperative Group of Treatment of Lymphoblastic Leukemia in Childhood (GBTLI), LLA-93 and LLA-99.

Standard Deviation	GBTLI LLA-93			GBTLI LLA-99		
	n=44	%	n=57	%
Sex: Male	17	38.6	24	42.1
Age at diagnosis (years)^a^	5.1±3.3		5.4±3.8	
Months withouth treatment^a^	154.6±26.5		89.9±20.8	
Risk: low risk	24	54.5	31	54.4
Prophylactic radiotherapy (12 Gy)	17	38.6	1	1.7
Radiation therapy (24 Gy)	3	6.8	3	5.3
Accumulated doses^a^				
Prednisone			1,169±160	
Dexamethasone	597±265		268±63	
Methotrexate	10,373±331		9,879±2,150	
Alkylating Agents	-		6,197±4,623	
6-mercaptopurine	36,145±4,936		32,898±539	

^a^Values expressed as mean (standard deviation).

With regard to the nutritional diagnosis, 22.8% of patients were considered overweight and 15.8% were considered obese, and there was no correlation between these conditions and previous exposure to radiation (*p* = 0.28). Body composition distribution showed that the lean mass levels and bone mineral content were higher in males, and fat mass levels and fat percentages were higher in females. Patients who had spent more time from the end of their treatment up to the time of the study had higher values of lean body mass, fat mass and percentage of fat and bone mineral content.

During the research, we observed two patients with osteonecrosis during treatment and two others who suffered a fracture of the forearm and tibia also in the same period.

Of the group of 79 children and adolescents under 20 years, three patients (2.9%) had low BMD and 16 (15.8%) were classified as at risk for low BMD. The characteristics of those three patients (two females, one male) with low BMD were: a mean age of 17±3 and an average of 11±3.6 years from the end of treatment up to the study. Two patients were classified as having common ALL and one as pre-B ALL; two were treated with protocol GBTLI LLA-93 and one with GBTLI LLA-99; two were considered as having low risk of relapse and one with high risk; no patient had infiltration of the CNS. The mean dose accumulated from chemotherapeutic agents and corticosteroids was: prednisone 2240±1584 mg/m^2^, dexamethasone 262±28 mg/m^2^, methotrexate 11713±1635 mg/m^2^, alkylating agents 2000 mg/m^2^ and 6-mercaptopurine (6-MP) of 32118-5007±5971 mg/m^2^; one patient was submitted to cranial radiotherapy with a dosage of 18 Gy. Two patients presented low BMD at the spine segment L1-L4 and one whole body BMD. As for body composition, the mean bone mineral content (BMC) level was 1757±376 g, while the mean fat percentage was 28.8±15.3%. Average fat mass was 13.6±8.8 kg and average lean mass was 30.6±3.3 kg.

Comparing the normal BMD group with the group at risk for low BMD, there was no significant difference linked to gender, race, age at diagnosis, current age, time outside therapy and pubertal stage nor to characteristics of the disease such as immunophenotype, initial leukocyte count, involvement of the central nervous system, risk of recurrence, chemotherapy treatment received, accumulated doses of prednisone, dexamethasone, methotrexate, alkylating agents, 6-MP and exposure to radiation therapy (Table 2). When matching lumbar spine segment L1-L4 and whole body BMD values with body composition for both groups, a significant difference was found in lean mass, which was lower in the group at risk for low lumbar spine L1-L4 and whole body BMD (*p*=0.043); no such difference was found with relation to other body composition variables. (Table 3).

**Table 2:** Rating distribution of the bone mineral density of whole body and lumbar spine L1-L4 of normal and risk groups of patients younger than 20 years old, regarding clinical features and related to treatment.

	Lumbar spine L1-L4 BMD			Whole body BMD		
	Normal	Risk	*p-*value	Normal	Risk	*p-*value
Males	25 (40.3%)	5 (33.3%)	0.83^a^	28 (38.9%)	3 (50%)	0.67^c^
Age at diagnosis (years)	4.0±2.6	4.4±2.6	0.62^b^	4.3±2.6	2.7±1.0	0.54^b^
Current age (years)	15.0±2.9	15.5±3.4	0.61^c^	15.1±3.0	14.7±3.1	0.72^c^
Months without treatment	107±33	111±44	0.68^b^	106±33	120±44	0.94^a^
Pubertal stage: pubertal	57 (91.9%)	13 (86.7%)	0.61^c^	66 (91.6%)	5 (83.3%)	0.44^c^
Pro-B immunophenotype	60 (96.7%)	14 (93.3%)	0.56^d^	69 (95.8%)	6 (100%)	0.49^d^
Initial White Blood Cell Count	33,049±47,097	33,767±53,261	0.95^b^	28,532±41,732	54,717±63,659	0.39^b^
CNS compromised	5 (8.1%)	-	0.57^c^	5 (6.9%)	-	1.0^c^
Low risk	36 (58.1%)	9 (60%)	1.0^a^	43 (59.7%)	4 (66.7%)	1.0^c^
Treatment						
GBTLI LLA-93	20 (32.2%)	6 (40%)	0.79^a^	23 (31.9%)	3 (50%)	0.39^c^
GBTLI LLA-99	42 (67.7%)	9 (60%)		49 (68.1%)	3 (50%)	
Accumulated dose (mg/m^2^)						
Prednisone	1147±121	1244±247	0.27^b^	1154±136	1307±323	0.27^b^
Dexamethasone	358±202	416±290	0.47^b^	353±201	446±333	0.69^b^
MTX	10197±1911	9842±765	0.26^b^	10141±1817	10656±1443	0.25^b^
Alkylates	6162±4635	4044±4057	0.19^b^	5943±4600	2000	0.19^b^
6-MP	34091±3461	33447±3070	0.51^b^	34122±3423	33338±3702	0.65^b^
Radiation Therapy	10 (16.1%)	4 (26.7%)	0.45^c^	11 (15.3%)	2 (33.3%)	0.26^a^

BMD: bone mineral density; CNS: central nervous system; GBTLI LLA: Brazilian Cooperative Group of Treatment of Lymphoblastic Leukemia in Childhood; MTX: methotrexate; 6-MP: 6-mercaptopurine; ^a^chi-square test; ^b^ *t* test; ^c^Fishers exact test; ^d^likelihood ratio test; *p*<0.05.

**Table 3:** Rating distribution of the bone mineral density of whole body and lumbar spine L1-L4 and body composition in relation to normal and risk groups in patients younger than 20 years old.

	BMD classification of lumbar spine L1-L4			BMD classification of whole body		
	Normal	Risk	*p-*value	Normal	Risk	*p-*value
BMD	1,022±172	890±188	0.011^a^	1,081±126	928±107	0.005^a^
Fat mass (kg)	17.3±8.8	13.6±7	0.139^a^	16.8±8.7	12.3±7.2	0.137^a^
Lean mass (kg)	35±8.5	29.7±7.4	0.031^a^	34.6±8.5	27.4±3.8	0.043^a^
% total fat	32.1±12	30.4±9.7	0.615^a^	31.7±11.8	29.1±12.2	0.536^a^
BMC (g)	2150±633	1919±637	0.209^a^	2146±629	1548±437	0.248^a^

^a^ *t* test; BMD: bone mineral density; p<0.05; BMC: bone mineral content; normal: values of z-score less than or equal to -2; risk: values of z-score between -1 and -2.

Of the 22 patients over the age of 20, eight (7.9%) had osteopenia, and there was no diagnosis of osteoporosis. When clinical characteristics of the normal BMD group and those of the osteopenia group were compared, there was no significant difference linked to gender, race, age at diagnosis, time without therapy, immunophenotype, initial leukocyte, involvement of the central nervous system, risk of recurrence, chemotherapy treatment received, accumulated doses by chemotherapeutic treatment of dexamethasone, methotrexate and 6-MP or completion of radiation therapy (Table 4). The group that presented femoral BMD osteopenia was older than the group with normal BMD (*p*=0.001) (Table 5).

**Table 4:** Distribution of the classification of bone mineral density of the whole body and femoral head of normal and osteopenia groups in patients aged 20 years or more in relation to clinical characteristics and related to treatment.

	BMD classification of lumbar spine L1-L4			BMD classification of femur		
	Normal	Osteopenia	*p-*value	Normal	Osteopenia	*p-*value
Males	6 (40%)	4 (57.1%)	0.65^a^	8 (42.1%)	2 (66.7%)	0.57^a^
Age at diagnosis (years)	10.1±3.7	8.1±3.8	0.25^b^	9.2±3.8	11.3±3.1	0.37^b^
Current age (years)	24.5±2.9	22.7±2.9	0.18^b^	23.5±3	26.7±0.6	0.001^b^
Months without treatment	149.6±38.2	166.3±27.2	0.31^b^	152.8±36.8	168±24	0.5^b^
Pro-B immunophenotype	12 (80%)	7 (100%)	0.1^c^	16 (84.2%)	3 (100%)	0.29^c^
Initial White Blood Cell Count	51,760±98,427.7	32,614.3±65,214.1	0.64^b^	52,163.2±93,391.9	4,533.3±3,477.5	0.39^b^
CNS compromise	3 (20%)	1 (14.3%)	1.0^a^	4 (21.1%)	-	1.0^a^
Low risk	4 (26.7%)	4 (57.1%)	0.34^a^	7 (36.8%)	1 (33.3%)	1.0^a^
Treatment GBTLI LLA-93	11 (73.3%)	6 (85.7%)	1.0^a^	15 (78.9%)	2 (66.7%)	1.0^a^
Accumulated dose (mg/m^2^)						
Dexamethasone	604±268	497±267	0.39^b^	580±265	503±322	0.65^b^
Methotrexate	9631±1050	10127±988	0.30^b^	9821±1010	9582±1408	0.71^b^
6-MP	33971±4233	36711±4904	0.19^b^	34851±4583	34793±5146	0.98^b^
Radiation Therapy	8 (53.3%)	2 (28.6%)	0.38^a^	9 (47.4%)	1 (33.3%)	1.0^a^

BMD: bone mineral density; *p*<0.05; ALL: acute lymphocytic leukemia; CNS: central nervous system; GBTLI LLA: Brazilian Cooperative Group of Treatment of Lymphoblastic Leukemia in Childhood; 6-MP: 6-mercaptopurine; ^a^Fishers exact test; ^b^ *t* test; ^c^likelihood ratio test.

**Table 5:** Distribution of the classification of bone mineral density of the lumbar spine L1-L4 and femur and body composition in relation to normal and osteopenia groups in patients aged 20 years or more.

	BMD classification of lumbar spine L1-L4			BMD classification of femur		
	Normal	Osteopenia	*p-*value	Normal	Osteopenia	*p-*value
BMD CL L1-L4	1,243±71	1,051±29	<0.001^a^	-	-	-
Femoral BMD	-	-	-	1.1±0.1	0.9±0.1	0.48^a^
Fat mass (kg)	28.0±15.5	17.9±12.8	0.15^a^	25.0±16.0	23.3±10.9	0.86^a^
Lean mass (kg)	41.7±9.1	42.2±8.9	0.89^a^	41.6±8.6	43.8±1.8	0.69^a^
% total fat	37.6±13.9	28±15.7	0.15^a^	34.7±15.8	33.7±5.0	0.91^a^
BMC (g)	2741±345	2458±420	0.10^a^	2652±364	2656±601	0.98^a^

BMD: bone mineral density; CL L1-L4: lumber spine, vertebrae L1-L4; BMC: bone mineral content; ^a^ *t* test; p<0.05.

## DISCUSSION

The majority of patients in this study had normal bone mineral density values in comparison to the reference population. This demonstrates that bone mass gain occurs with increasing age and that treatment does not influence the process.^9^ ^.^ ^11^ In our research, three patients had low BMD. This finding varies 5-85% for populations of the same age and gender in several studies, and depends on the characteristics of the patients, the method used for BMD measurement and the way it is interpreted.^12^ ^.^ ^13^

The first two years after the end of ALL treatment is the most critical period for bone loss, with recovery and progressive increase of bone mass taking place after that time.^13^ Therefore, data suggests that this population had no risk for the loss of BMD. Some studies showed similar results: the larger the elapsed time between the end of therapy and the younger the patient at the beginning of treatment, the greater the chance to recover the loss of BMD and acquire adequate bone mass.^14^ ^.^ ^15^ The clinical implication of low BMD in these patients is still uncertain, especially as it pertains to the risk of fractures.^16^ The frequency of low BMD in this study was 2%, compared to the prevalence of 11-18.5% of fractures in the literature.^17^ ^.^ ^18^ In the same fashion, a lower prevalence of osteonecrosis was found in our study.^19^ ^.^ ^20^ These results are attributed to possible subdiagnoses and the absence of active search for osteonecrosis in asymptomatic patients.

Vitamin D levels were below normal in 23 patients (23.4%). Some analyses with Brazilian children and adolescents showed that 60-70% of them had insufficient levels of vitamin D, with inadequate intake of this vitamin and calcium. Therefore, the lack of these nutrients may not be related to the treatment received.^21^ ^,^ ^22^ ^,^ ^23^ ^,^ ^24^ However, some studies have shown that ALL treatment increases the incidence of vitamin D deficiency, which potentially increases the risk of low bone mineral density.^25^

Although current densitometry standards define low BMD (for people under 20), as that which presents z-scores under -2 SDs in comparison to the reference for age and gender,^8^ ^.^ ^10^ in the present study we observed that the risk group (z-score between -1.1 and -1.9), considered by the literature as normal BMD, in reality presented significantly lower values of BMD. Therefore, this population could benefit from early diagnoses and implementation of preventive measures aimed at detecting bone toxicity in the long term and avoiding greater bone mass losses. Some of these measures are based on changes in life habits, encouraging physical activity and eating a vitamin D and calcium-rich diet in order to ensure the stability of bone mass gain.^15^ ^.^ ^26^

With regard to to body composition, in that same group, there was also a difference between the amount of lean mass and lumbar spine and whole body BMD. Another study with a similar population also found this difference.^27^ This reinforces the fact that lean mass is one of the most important predictive factors of bone mass.^16^ ALL treatment may cause changes in body composition, such as obesity, decrease in lean body mass, growth deficits and changes in BMD. The relationship between bone mass, anthropometric data and lean mass may vary according to age, gender and growth rate, as well as nutritional status, often measured by the BMI, which is also a determinant of bone mass.^16^

We also analysed patients older than 20 years of age in contrast with the group with osteopenia and with the group with normal lumbar spine and femoral BMD. There were no differences found related to the disease or to the accumulated doses of corticosteroids and chemotherapy agents related to lumbar spine L1-L4 and femoral head BMD. Only the average lumbar spine L1-L4 BMD was significantly lower in the osteopenia group in comparison to the normal BMD group. This may be due to the fact that the lumbar spine vertebrae, which are more sensitive to toxic bone factors, are composed of trabecular bone, more metabolically active than cortical bone. The basis for the establishment of bone mass in adults is initiated in childhood and adolescence. Bone metabolism and BMD development are influenced by countless factors. Any interference in this process causes a deficit in the acquisition of bone mass, impairing the acquisition of peak bone mass, an important determinant of the risk of fractures in adulthood.^28^

For the first time, the bone toxicity of patients treated for ALL was analysed in accordance with two Brazilian protocols coming from a single institution. Understanding the reality of Brazilian patients treated for childhood ALL with regard to late bone toxicity allows for the development of a longitudinal follow-up focused on this population, with its peculiar characteristics, providing good opportunity for future interventions. In Brazil, so far, there is no national guideline for tracing late effects of antineoplastic treatment, or their impact on BMD. Such a guideline would provide subsidies for the creation of risk scores based on characteristics of the Brazilian ALL survivor population, with respect to the treatment received and the bone development commonly associated with our population.

This research had some limitations: there was a significant loss of patients - 53% died and the rest were excluded by criteria), with a decrease in the study sample and a possible impact on the results presented. As this was a cross-sectional study, it was not possible to verify the association of cause and effect between the bone toxicity and ALL treatment. Furthermore, the *software* used had no reference data based on the Brazilian pediatric population with specific BMD values for comparison. Finally, there was no healthy children population available as a control for data comparison, and thus we were not able to determine whether there were other factors that influence bone toxicity other than exposure to treatment.

It can be concluded that the impact of the treatment in accordance with Brazilian protocols GBTLI LLA-93 and ­LLA-99 on survivors’ BMD in the long term was low, and 2.9% of patients had low BMD.

Through this study, it was possible to define a risk group for low BMD, made up of 15.8% of the patients, whose values of whole body and lumbar spine L1-L4 BMD were significantly lower than the population studied, values that are in fact considered normal (below 2 SD) in the literature. This group may be benefit from preventive actions against bone mass loss and the development of protocols for longitudinal follow-up and detection of bone toxicity. In the population studied, the occurrence of fractures (2%) and osteonecrosis (2%) was uncommon, however no active search for osteonecrosis was performed on asymptomatic patients. Lean mass was related to whole body and lumbar spine L1-L4 BMD values in patients under 20 years of age. The higher the values of lean mass, the greater the BMD. The present study has suggested a positive association between BMD and lean mass, confirming its importance in the development of bone mass.
